# A Rare Case of Bilateral Hip Tumoral Calcinosis With Intertrochanteric Fracture Treated by Closed Reduction Internal Fixation (CRIF) Using a Proximal Femoral Nail

**DOI:** 10.7759/cureus.32575

**Published:** 2022-12-15

**Authors:** Shirsha Ray, Vellanki Sravan, Rushikesh Abhyankar, Swaroop Solunke

**Affiliations:** 1 Orthopaedics, Dr. D. Y. Patil Medical College, Hospital & Research Centre, Pune, IND

**Keywords:** rare, proximal femoral nail, orthopaedics, intertrochanteric fracture, tumoral calcinosis

## Abstract

Tumoral calcinosis, also referred to as Teutschländer disease is a rare familial disorder characterized by painless, periarticular lumps or masses. Large amorphous calcific concentrations that surround the joints are the defining feature. These lesions have fibrous septa that divide them into lobules and may often show fluid/calcium levels (milk of calcium/suspended hydroxyapatite crystals).

In our case report, a 24-year-old male came to the outpatient department (OPD) with complaints of pain over the right hip for two days and swelling over both hips for six years. He was apparently well two days prior to the hospital visit, when he met with a road traffic accident, falling off his bike. A plain radiograph of the pelvis and bilateral hip joints along with cross-table lateral views were done, which depicted a typical appearance of amorphous and multilobulated ("cloud-like") calcifications located in a periarticular distribution in both hips along with an intertrochanteric fracture in the right hip. A CT scan, done for better delineation of the calcific masses and fracture pattern, showed no obvious erosion or osseous destruction by the adjacent soft-tissue masses. Laboratory investigations revealed a serum calcium level of 8.8 mg/dl and a serum phosphorous level of 6.0 mg/dl.

The patient was taken up for surgery after routine pre-operative investigations and a pre-anaesthesia check-up. Closed reduction internal fixation (CRIF) was done using a proximal femoral nail (PFN). A biopsy of the soft-tissue masses was sent for histopathology, which was suggestive of lobules of calcific material surrounded by histiocytic giant cells. The patient responded well to the treatment with no residual discharge from the incision site, and his treatment was continued with phosphate binders as prescribed by the endocrinologist.

## Introduction

Tumoral calcinosis is an uncommon disorder causing calcium deposition in the soft tissues around joints and outside the joint capsule. They often appear in people receiving renal dialysis (0.5-3%) [1]. Clinically sometimes referred to as hyperphosphatemic familial tumoral calcinosis (HFTC), this condition is frequently brought on by genetic abnormalities in the genes that control the body's phosphate physiology, resulting in hyperphosphatemia (an excess of phosphate). The most well-known human mutation-prone genes include *FGF-23* [2], Klotho (*KL*) [3], or *GALNT3* [4].

According to the term, tumoral calcinosis (calcium deposition) resembles a tumor. Since they don't have dividing cells, they are not real neoplasms. They are just inorganic calcium deposits with serum exudate. The most prevalent age groups to be impacted are children and teenagers (6 to 25 years) [5]. Instead of discomfort, the accumulations' symptoms include edema around the joints. They tend to gradually become bigger, ulcerate the skin above them, and protrude. The most frequent sites are the elbows, shoulders, and hips [6]. Results of the laboratory analysis often show hyperphosphatemia and normal serum calcium levels [7].

## Case presentation

**Figure 1:**
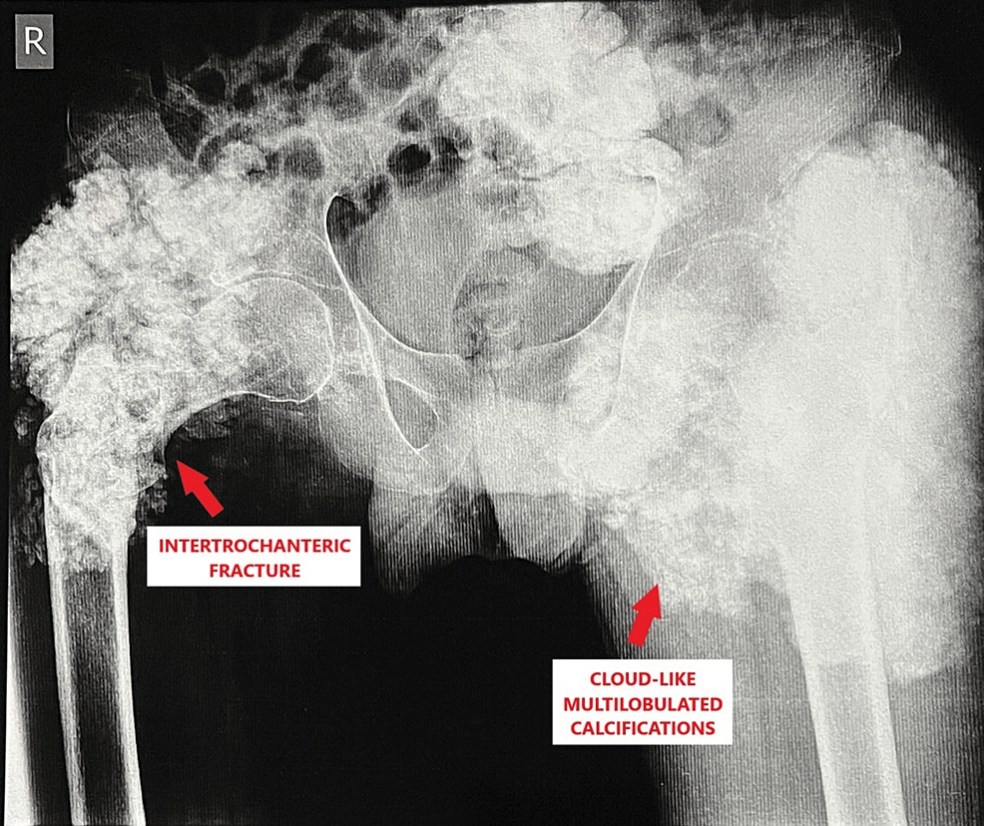
Plain radiograph showing pelvis and both hip joints.

**Figure 2:**
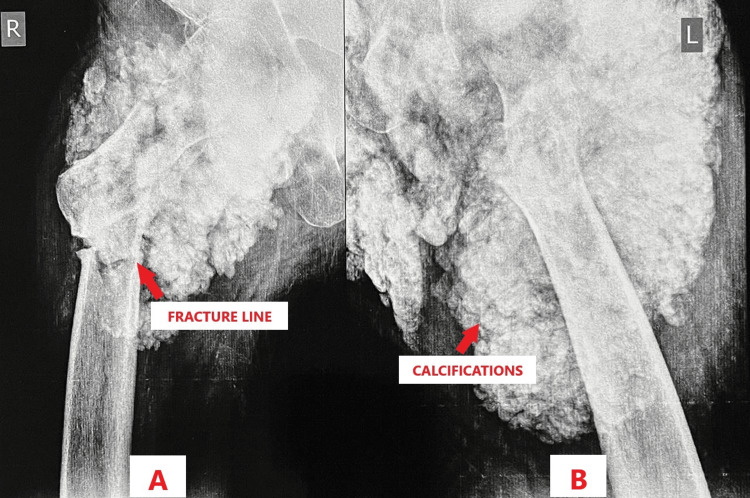
Radiograph of cross-table lateral views of the right and left hip. 2A: Right cross-table lateral view showing the fracture line. 2B: Left cross-table lateral view showing peri-articular calcific depositions.

In our case report, a 24-year-old male came to the outpatient department (OPD) with complaints of pain over the right hip for two days and swelling over both hips for six years. He was apparently well two days prior to the hospital visit, when he met with a road traffic accident, falling off his bike following which he developed pain in his right hip which was acute in onset, continuous in nature, aching in character, moderate in severity, aggravated on movement and relieved on rest, immobilization and pain medication. Additionally, he gave a history of swelling in both hips for six years which was insidious in onset and had been progressively increasing in nature. A plain radiograph of the pelvis and bilateral hip joints along with cross-table lateral views were done, which depicted a typical appearance of amorphous and multilobulated ("cloud-like") calcifications located in a periarticular distribution in both hips along with a stable two-part intertrochanteric fracture in the right hip (Type 1 according to Boyd and Griffin classification) [8], as shown in Figure 1 and Figures 2A, 2B.

**Figure 3:**
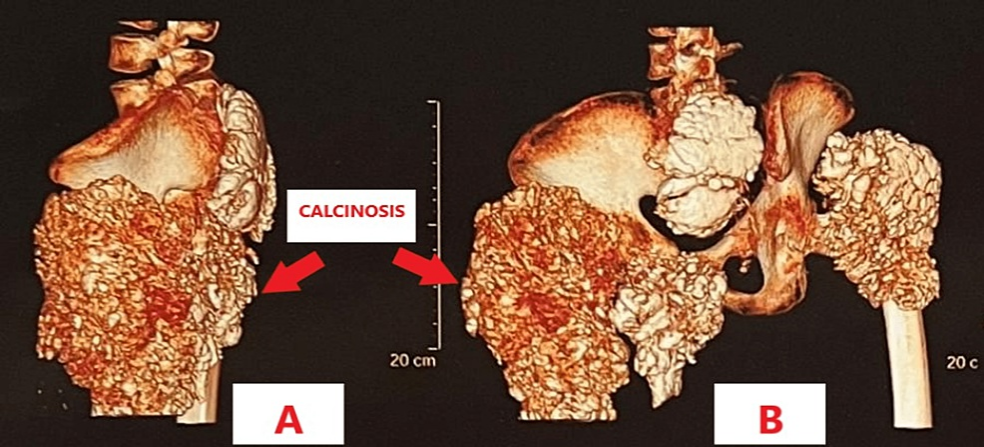
3D CT scan of pelvis with both hips showing calcinosis. 3A: Lateral View 3B: Posteroanterior (PA) View

A CT scan, done for better delineation of the calcific masses and fracture pattern, showed no obvious erosion or osseous destruction by the adjacent soft-tissue masses as shown in Figure 3.

**Figure 4:**
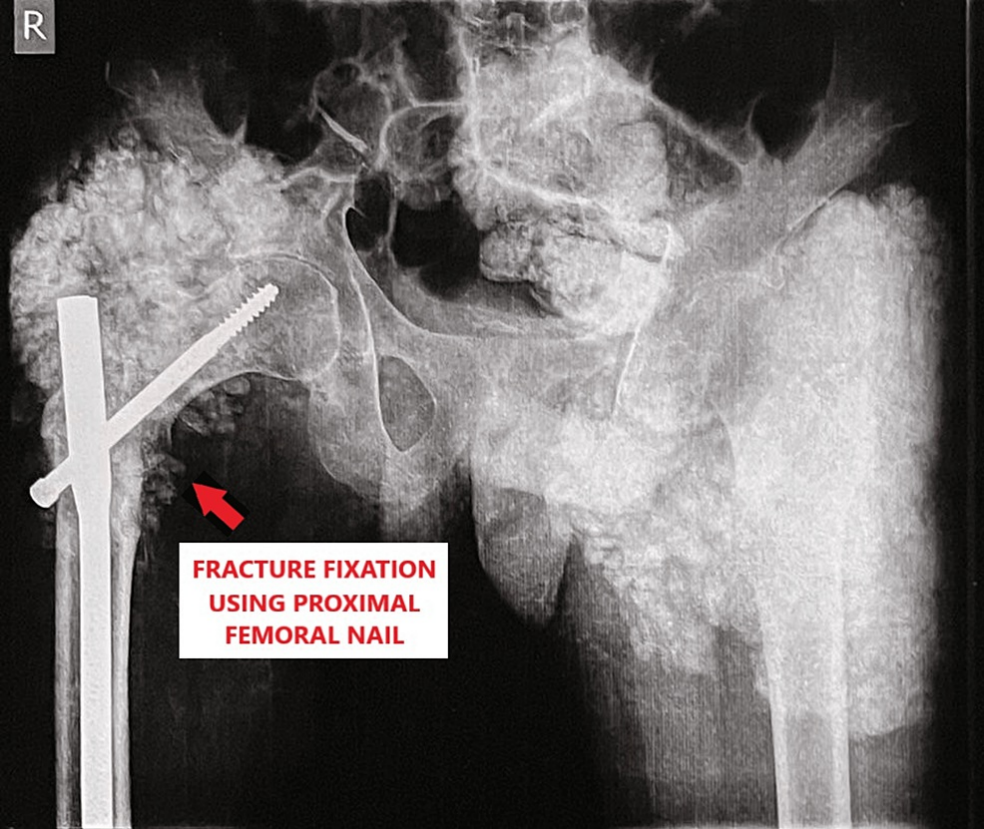
Post-operative radiograph showing fracture fixation using a proximal femoral nail.

Laboratory investigations revealed a serum calcium level of 8.8 mg/dl and a serum phosphorous level of 6.0 mg/dl. The patient was taken up for surgery after routine pre-operative investigations and a pre-anaesthesia check-up. Closed reduction internal fixation (CRIF) was done using a proximal femoral nail (PFN) as shown in Figure 4.

**Figure 5:**
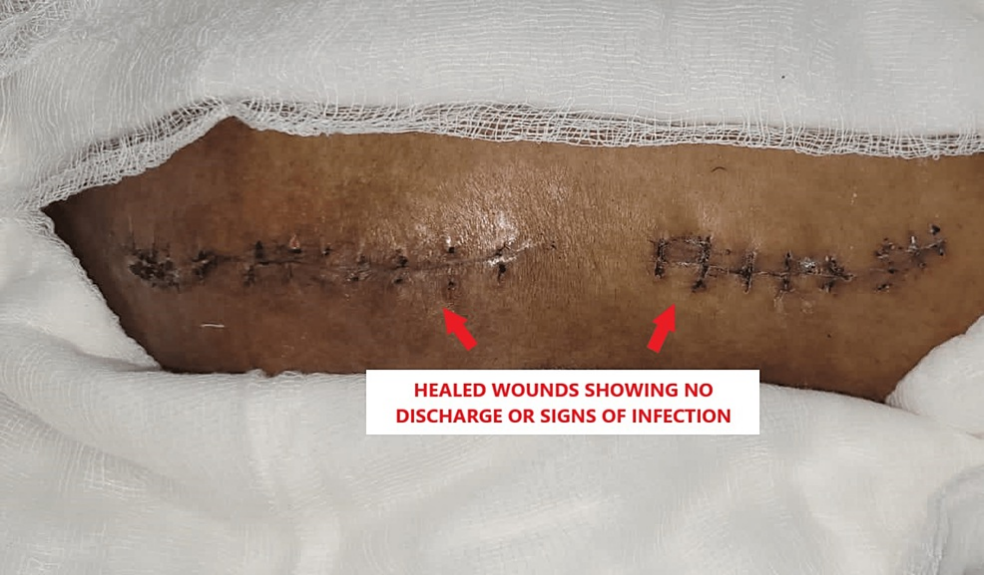
Post-operative day 18 wound after suture removal.

A biopsy of the soft-tissue masses was sent for histopathology, which was suggestive of lobules of calcific material surrounded by histiocytic giant cells. Post-surgery, dressings were done on post-operative days 2, 8, and 12while sutures were removed on day 18 as shown in Figure 5.

Non-weight-bearing walking was advised for six weeks along with range-of-motion exercises as per tolerance. The patient responded well to treatment with no residual discharge from the incision site and continued with phosphate binders as prescribed by the endocrinologist.

## Discussion

For a long time, the clinico-pathological concept of tumoral calcinosis has been contentious. It is best described as a condition with calcium depositions in the peri-articular regions affecting mainly shoulder, elbow, and hip joints.

In our case, a 24-year-old male with a history of bilateral hip tumoral calcinosis presented with a post-traumatic intertrochanteric fracture in the right hip. The main challenge faced in fracture fixation was an obstruction to the entry point of the proximal femoral nail and difficult visualisation under C-arm fluoroscopy due to the multilobulated masses around the hip joint. Once fracture fixation was done, the aim of the surgery was to ensure tight closure of the surgical wound to prevent discharge secondary to calcinosis to facilitate optimal wound healing. 

Post-operatively, the patient was prescribed phosphate binders by the endocrinologist and advised range-of-motion exercises as per tolerance. Non-weight-bearing walking was advised for six weeks as a precautionary measure to avoid early implant failure. Sutures were removed on post-operative day 18 showing no signs of infection or discharge. Overall, the patient responded well to treatment and the post-operative period was uneventful.

## Conclusions

The present case demonstrated amorphous and multilobulated ("cloud-like") calcifications located in a periarticular distribution in both hips along with an intertrochanteric fracture in the right hip. A closed reduction internal fixation (CRIF) procedure was done using a proximal femoral nail (PFN) to treat the fracture. The patient responded well to the treatment and was continued on phosphate binders as prescribed by the endocrinologist.

Fracture fixation in a case of tumoral calcinosis is a challenging procedure mainly due to the obstruction of the visual field under C-arm fluoroscopy and the bulk of calcium depositions encountered during surgery. Detailed pre-operative planning to define the expected outcome is the key to successful management in such cases.
